# Supplementary material for: SORFPP: Enhancing rich sequence-driven information to identify SEPs based on fused framework on validation datasets
Source: PLoS One. 2025 Apr 28;20(4):e0320314. doi: 10.1371/journal.pone.0320314 (PMC12036913; doi:10.1371/journal.pone.0320314)
Supplement: S1 Table — . (DOCX) [file pone.0320314.s001.docx]

Table S1. Explanation of three fusion networks.

| Model | Explanation |
| --- | --- |
| Attention_BiLSTM (ABiLSTM) | The Attention_BiLSTM model incorporated an attention mechanism into the BiLSTM model to perform relation extraction tasks. ABiLSTM model streamlined the experimental process by eliminating the requirement for complex feature engineering. |
| Attention_BiGRU (ABiGRU) | The Attention_BiGRU model applied an attention mechanism to the outputs of BiGRU by computing attention weights for each time step. Thus, the ABiGRU model was capability of effectively processing long sequences and capturing crucial local information. |
| CNN_LSTM (CLSTM) | The CNN-LSTM model initially extracted local features from the input features by sliding convolutional kernels over the sequence. Then, these extracted features were fed into an LSTM network. The LSTM network captured long-term dependencies by maintaining a hidden state and processing the feature sequence progressively over time steps. |

Note: Attention_BiLSTM (ABiLSTM); BiGRU_Attention (ABiGRU); CNN_LSTM (CLSTM)

Fig S1. ABiLSTM Network.

Fig S2. ABiGRU Network.

Fig S3. CLSTM Network.
