# Supplementary figures and images for: SORFPP: Enhancing rich sequence-driven information to identify SEPs based on fused framework on validation datasets
Source: PLoS One. 2025 Apr 28;20(4):e0320314. doi: 10.1371/journal.pone.0320314 (PMC12036913; doi:10.1371/journal.pone.0320314)

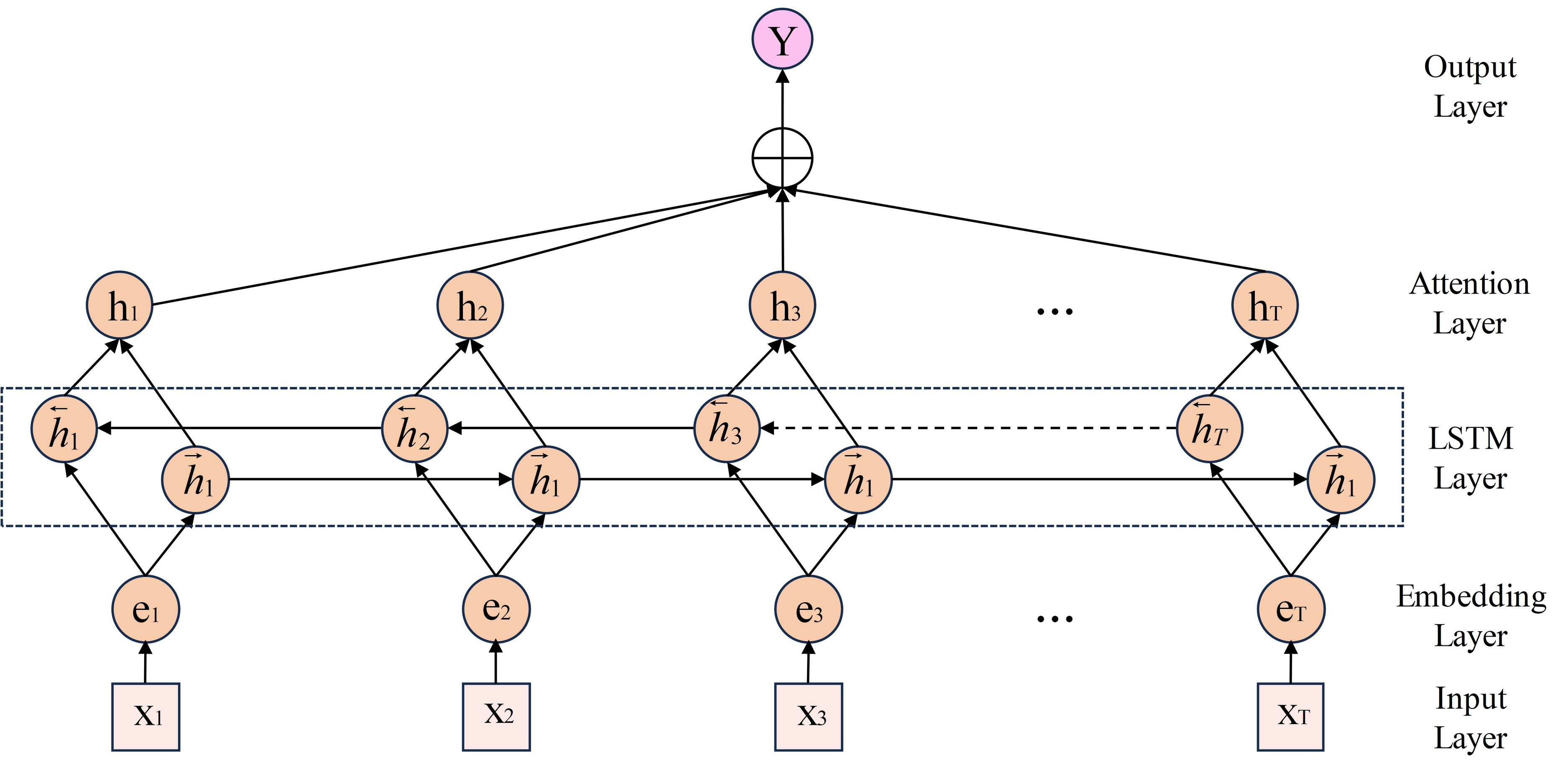

Supplement: S1 Fig — (TIF) [file pone.0320314.s002.tif]

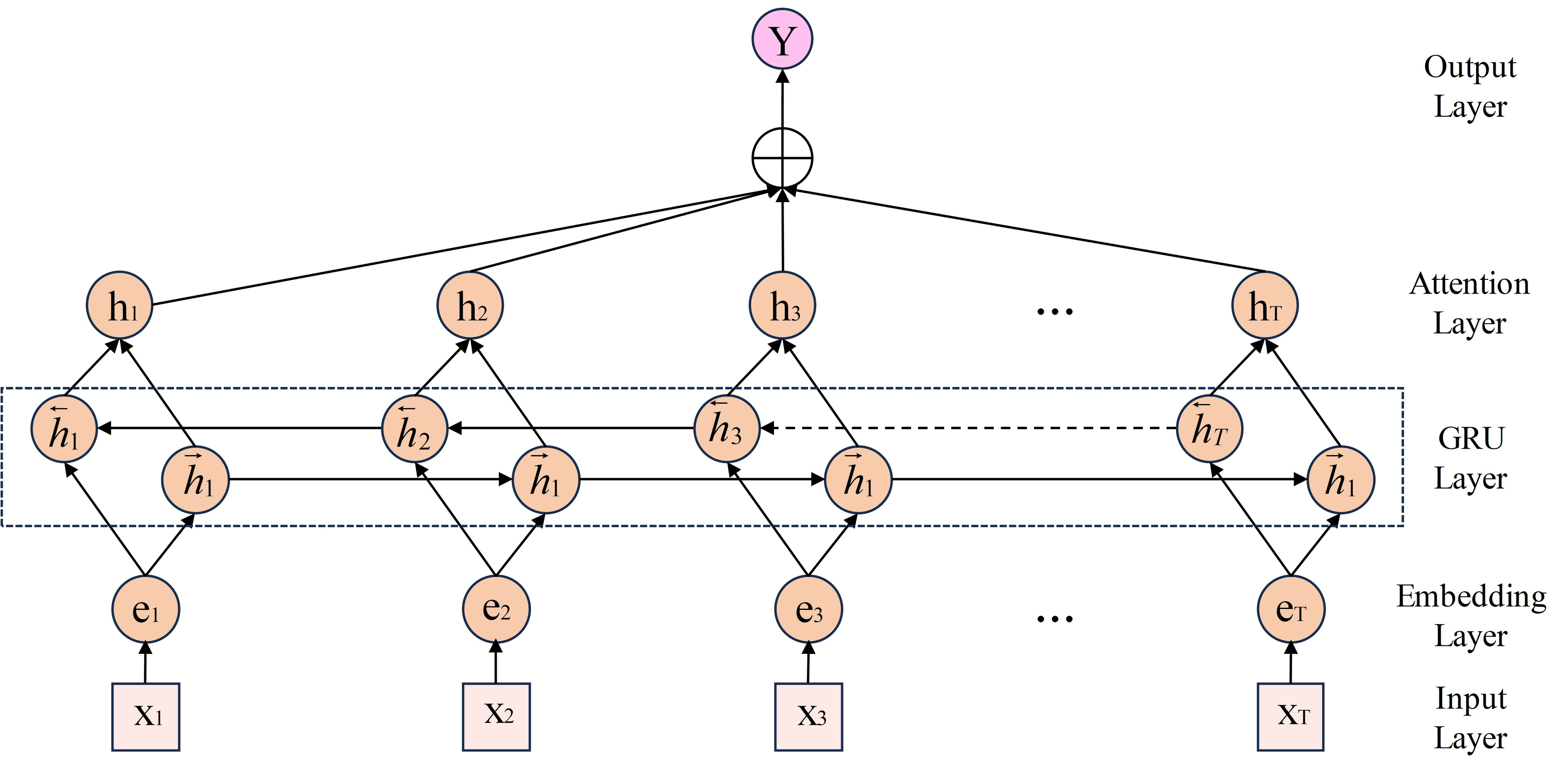

Supplement: S2 Fig — (TIF) [file pone.0320314.s003.tif]

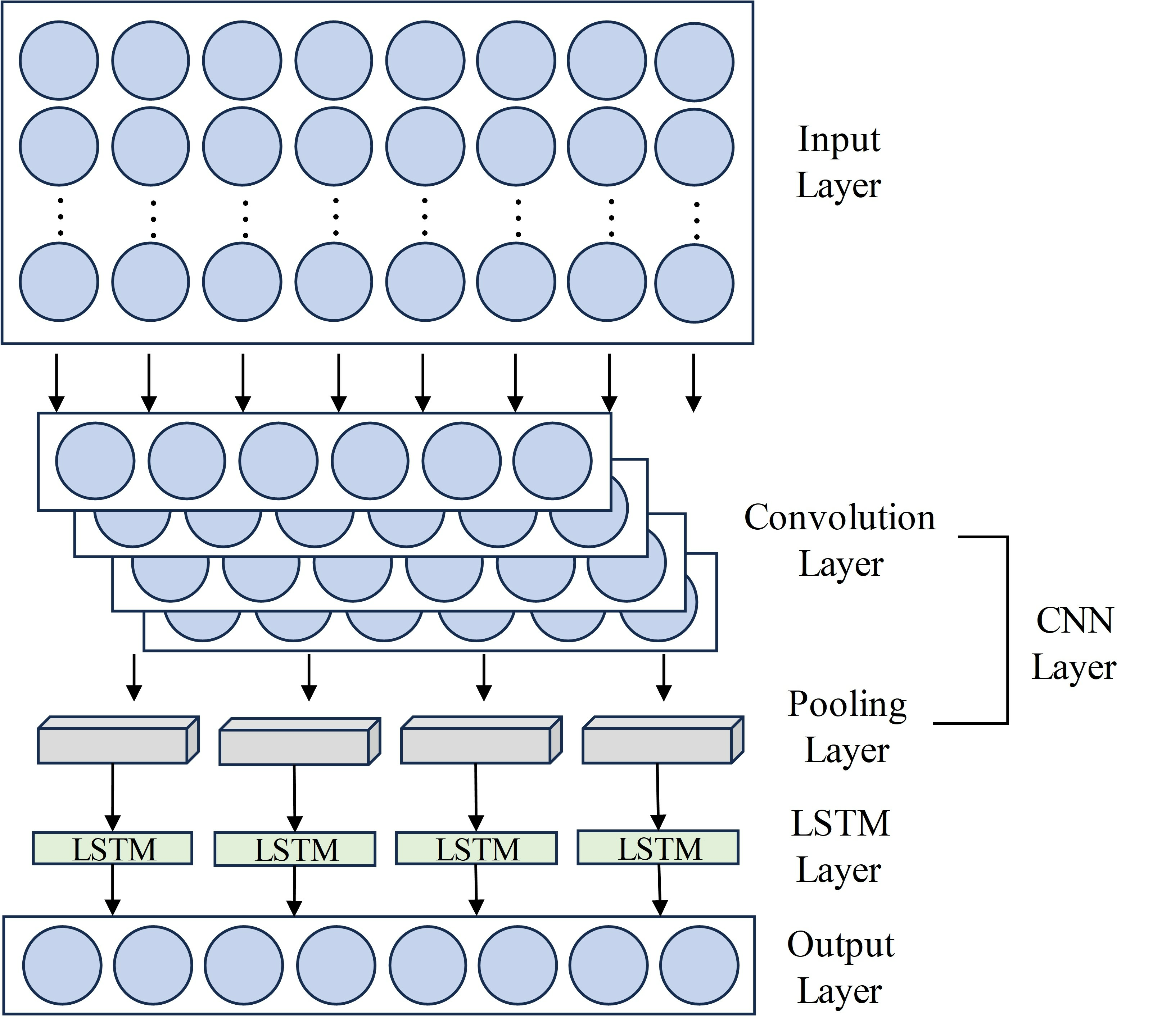

Supplement: S3 Fig — (TIF) [file pone.0320314.s004.tif]
