# Supplementary material for: SORFPP: Enhancing rich sequence-driven information to identify SEPs based on fused framework on validation datasets
Source: PLoS One. 2025 Apr 28;20(4):e0320314. doi: 10.1371/journal.pone.0320314 (PMC12036913; doi:10.1371/journal.pone.0320314)
Supplement: Attachment — Submitted filename: response_to_reviewers-SORFPP_auresp_2.docx [file pone.0320314.s007.docx]

**Response to the reviewers**

Dear Editor and Reviewers:

Thank you for taking out of your busy schedule to review the manuscript. Now we have carefully revised and replied to the manuscript for this version. The revision instructions are as follows:

**To Journal requirements:**

1. **Answer:** Thanks for your suggestion. Each project funder has been identified and their specific research role has been detailed in the CRediT authorship contribution statement section.

**To Reviewer: 1**

Thank you for evaluating the revised manuscript. We sincerely appreciate Reviewer #1's acknowledgment that our responses have adequately addressed the previous comments, and are gratified to learn that the revisions meet the expectations. The expert feedback has been instrumental in refining this study. Substantial improvements have been implemented through additional experiments, which significantly enhanced the quality of the paper.

Should there be any remaining aspects requiring clarification, we remain fully committed to further refinements. Once again, we extend our deepest gratitude to the editorial team and reviewers for dedicating your valuable time and expertise to improve this work.

**To Reviewer: 2**
**Question 1. Why do authors use Categorical Boost instead of Ada Boost for instance?**

**Answers:** Thank you for your insightful question regarding our choice of Categorical Boost over AdaBoost. The selection of the CatBoost model was based on three considerations: the scale of the dataset, the feature methods, and experimental comparisons.

Firstly, the dataset used in this experiment is large in size. CatBoost is specifically designed to handle categorical features without extensive preprocessing, making it particularly suitable for datasets with a large number of categorical variables. In contrast, AdaBoost typically relies on decision trees, which do not locally optimize the encoding of categorical features, potentially leading to suboptimal splits and increased training complexity. Moreover, the feature calculation methods used in this experiment may produce sparse values. CatBoost incorporates regularization techniques, which help to mitigate overfitting. Finally, through experimental comparisons, CatBoost has shown superior performance across various evaluation metrics compared to AdaBoost.

| Feature | Model | ACC | PRE | AUC | MCC | F1 | SN | SP |
| --- | --- | --- | --- | --- | --- | --- | --- | --- |
| AN Feature (Human) | **CatBoost** | **0.876** | **0.875** | **0.957** | **0.753** | **0.878** | **0.881** | **0.872** |
|  | AdaBoost | 0.866 | 0.871 | 0.945 | 0.733 | 0.868 | 0.870 | 0.873 |
| AN Feature (Mouse) | **CatBoost** | **0.867** | **0.859** | **0.948** | **0.734** | **0.864** | **0.869** | **0.865** |
|  | AdaBoost | 0.850 | 0.841 | 0.941 | 0.700 | 0.849 | 0.856 | 0.851 |
| AN Feature (Rat) | **CatBoost** | **0.822** | **0.802** | **0.913** | **0.645** | **0.818** | **0.836** | **0.810** |
|  | AdaBoost | 0.799 | 0.765 | 0.880 | 0.598 | 0.790 | 0.814 | 0.788 |

**Question 2. Figure 3 colors are not clear. Can you may be make it with fewer colors?**

**Answers:** Thank you for your valuable feedback regarding Figure 3. We appreciate your suggestion to improve the clarity of colors. To address this concern, we have reduced the number of colors used in the figure while maintaining the distinction among different elements. This adjustment enhances readability and ensures better visual contrast.

**Fig. 3. Comparison results of nucleotide and amino acid features on three datasets.**

**Please do not hesitate to contact us if there are any question. Thanks again to the reviewers and editors for your hard work! Best wishes to you!**
